# Salidroside ameliorates sepsis-induced acute lung injury and mortality *via* downregulating NF-κB and HMGB1 pathways through the upregulation of SIRT1

**DOI:** 10.1038/s41598-017-12285-8

**Published:** 2017-09-20

**Authors:** Kuo-Cheng Lan, Sung-Chuan Chao, Hsiao-Yi Wu, Chia-Lien Chiang, Ching-Chia Wang, Shing-Hwa Liu, Te-I. Weng

**Affiliations:** 1 Department of Emergency Medicine, Tri-Service General Hospital, National Defense Medical Center, Taipei, Taiwan; 2 Department of Surgery, National Taiwan University Hospital Hsin-Chu Branch, Hsin-Chu, Taiwan; 3 Department of Forensic Medicine, College of Medicine, National Taiwan University, Taipei, Taiwan; 4 Departments of Pediatrics, National Taiwan University Hospital, Taipei, Taiwan; 5 Institute of Toxicology, College of Medicine, National Taiwan University, Taipei, Taiwan; 6 Departments of Emergency Medicine, National Taiwan University Hospital, Taipei, Taiwan

## Abstract

Sepsis is a life-threatening medical condition. Salidroside, a substance isolated from *Rhodiola rosea*, possesses antioxidant and anti-inflammatory properties. The effect and mechanism of salidroside on sepsis-induced acute lung injury still remains to be well clarified. Here, we investigated the effect and mechanism of salidroside on septic mouse models and explored the role of salidroside-upregulated SIRT1. Salidroside inhibited the inflammatory responses and HMGB1 productions in bacterial lipopolysaccharide (LPS)-treated macrophages and mice. Salidroside could also reverse the decreased SIRT1 protein expression in LPS-treated macrophages and mice. Salidroside also alleviated the sepsis-induced lung edema, lipid peroxidation, and histopathological changes and the mortality, and improved the lung PaO_2_/FiO_2_ ratio in cecal ligation and puncture (CLP)-induced septic mice. Salidroside significantly decreased the serum TNF-α, IL-6, NO, and HMGB1 productions, pulmonary inducible NO synthase (iNOS) and phosphorylated NF-κB-p65 protein expressions, and pulmonary HMGB1 nuclear translocation in CLP septic mice. Moreover, sepsis decreased the SIRT1 protein expression in the lungs of CLP septic mice. Salidroside significantly upregulated the SIRT1 expression and inhibited the inflammatory responses in CLP septic mouse lungs. These results suggest that salidroside protects against sepsis-induced acute lung injury and mortality, which might be through the SIRT1-mediated repression of NF-κB activation and HMGB1 nucleocytoplasmic translocation.

## Introduction

Sepsis is a life-threatening medical condition characterised by dysregulated inflammation, dysfunctional blood coagulation, and multiple organ injuries^1^. The annual prevalence of sepsis worldwide is estimated at 19 million^2^. Despite modern antimicrobials, severe sepsis-related mortality remains high at 20–30%^1,2^. New therapeutic targets are urgently required to improve the survival outcomes of septic patients. Sepsis has a biphasic inflammatory process: an early phase characterised by proinflammatory cytokines such as tumour necrosis factor (TNF) and interleukins (IL), and a late phase mediated by an inflammatory high-mobility group box 1 (HMGB1) protein^3^. The balance between pro-inflammatory and anti-inflammatory pathways is critical in the survival and well-being of patients^4^. Severe inflammation can be deleterious, resulting in multiple organ dysfunction, including acute lung injury. acute lung injury is the most common organ injury in sepsis and causes severe lung inflammation^5^. Patients with sepsis-induced acute lung injury had greater illness severity and organ dysfunction^6^. Despite extensive investigation for many years, acute lung injury remains a serious problem in intensive care.

HMGB1 is critical in the pathogenesis of sepsis. HMGB1 is known to store in the nucleus. Bacteria lipopolysaccharide (LPS) can cause HMGB1 acetylation, resulting in the localization of the protein to the cytosol^7^. This translocation causes the accumulation of cytosolic HMGB1, leading to its secretion through a vesicle-mediated secretory pathway in monocytes and macrophages^7^. Extracellular HMGB1 is a late mediator of sepsis and acts as a key regulator in acute and chronic inflammation^3,8^. Inhibition of HMGB1 secretion attenuates systemic inflammatory response syndrome and sepsis-induced organ injury (Wang *et al*. 2008). In addition, nuclear factor (NF)-κB is a critical transcription factor for the maximal expression of numerous cytokines involved in the pathogenesis of acute lung injury^9^. On the other hand, SIRT1, a NAD+-dependent deacetylase, is constitutively expressed in most cells and is involved in signaling pathways regulating the cellular life span and oxidative stress responses^10^. SIRT1 has been shown to inhibit NF-κB transcriptional activity through the de-acetylation of the p65 subunit, leading to reduce the inflammatory cytokine production and activation^10,11^.

Adaptogens are known to be the botanical species that may help to maintain the normalizing bodily functions and processes. In traditional folk medicine, *Rhodiola rosea* is used as an adaptogen for enhancing resistance to fatigue, stimulating the nervous system, and preventing high-altitude sickness^12^. Salidroside, an 8-O-b-d-glucoside of tyrosol, is the main bioactive component of *R. rosea* ^13^. Salidroside possesses various pharmacological properties and exerts antioxidative and antiinflammatory effects^14,15^. Salidroside exerts protective effects on chronic intermittent hypoxia-induced, Fas-dependant, and mitochondria-dependant apoptotic pathways in the mouse hearts^16^. Salidroside protected septic rats from acute lung injury by upregulating peroxisome proliferator-activated receptor γ expression and attenuating LPS-activated NF-κB signaling^17^. Salidroside also improved the survival and suppressed the proinflammatory responses during sepsis^18^. However, the mechanism through which salidroside confers protection against acute lung injury remains elusive. Moreover, resveratrol has been found to improve septic liver injury through a SIRT1-regulated HMGB1 acetylation pathway^19^. Therefore, we hypothesized that SIRT1 signaling pathway might be involved in the therapeutic effect of salidroside on sepsis-induced acute lung injury. We used a bacterial lipopolysaccharide (LPS)-induced systemic inflammation mouse model and a cecal ligation and puncture (CLP)-induced sepsis mouse model to investigate the protective effects of salidroside and explore the possible underlying action mechanisms.

## Results

### Effects of salidroside on inflammatory responses in LPS-treated macrophages and LPS-induced systemic inflammation mouse model

**Figure 1:**
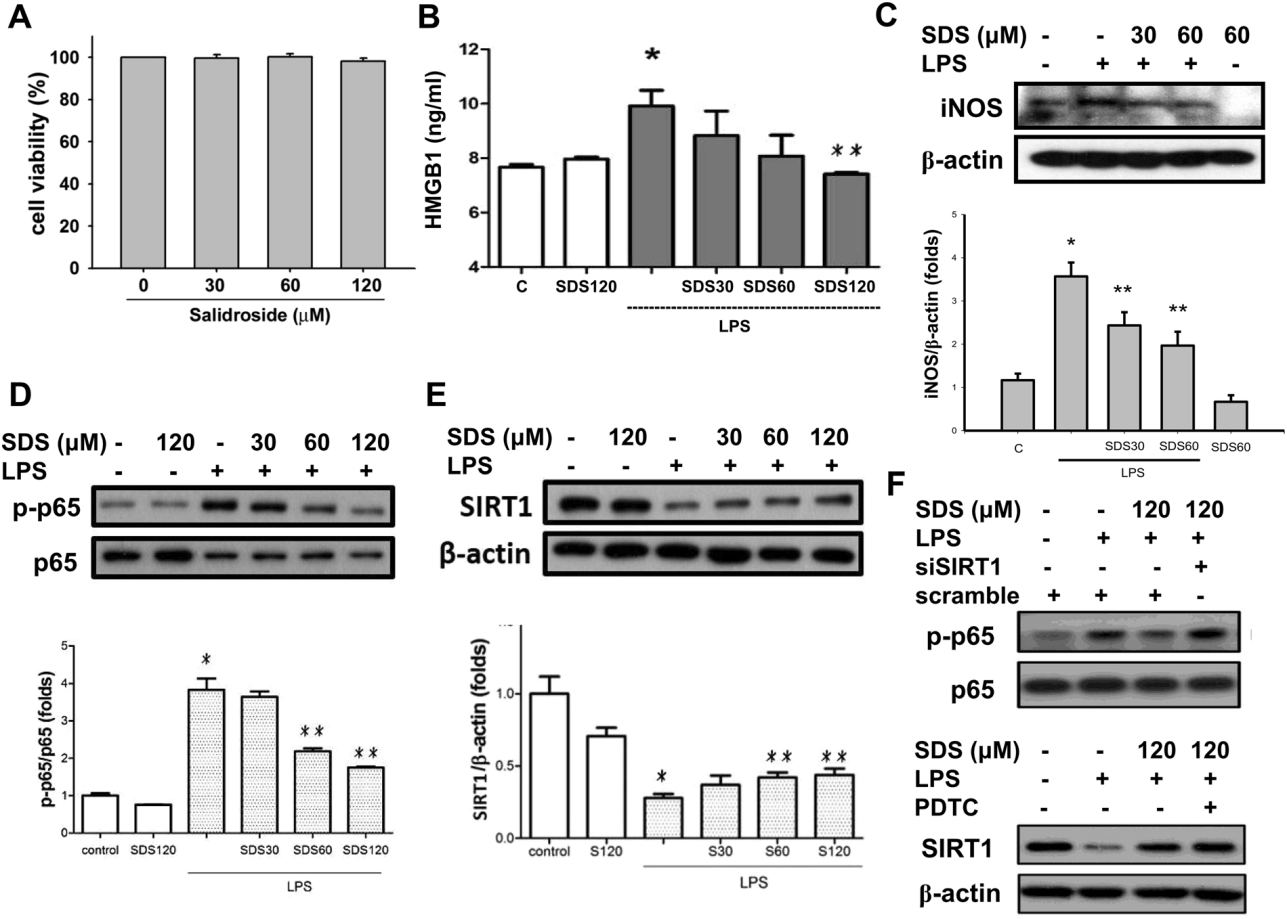
Salidroside inhibited LPS-induced inflammatory responses in macrophages. (**A**) Cell viability was detected in RAW264.7 macrophages with or without salidroside (30, 60, and 120 μM) treatment for 24 h. Moreover, RAW264.7 macrophages were stimulated with LPS (1 μg/ml) in the presence or absence of salidroside (30, 60, and 120 μM) for 16 h (**B**), HMGB1 production; (**C**) iNOS protein expression; (**E**) SIRT1 protein expression) or 1 h (**D**), NF-κB-p-65 phosphorylation). The HMGB1 levels were determined by an ELISA kit. (**F**) To clarify the relationship between SIRT1 and NF-κB signals, the siRNA-SIRT1 transfection and a selective NF-κB inhibitor pyrrolidine dithiocarbamate (PDTC) were used and the NF-κB-p-65 phosphorylation and SIRT1 protein expression were detected. The protein expressions were determined by Western blot. Data are presented as means ± SEM (n = 4). *P < 0.05 as compared with control. **P < 0.05 as compared with LPS alone.

**Figure 2:**
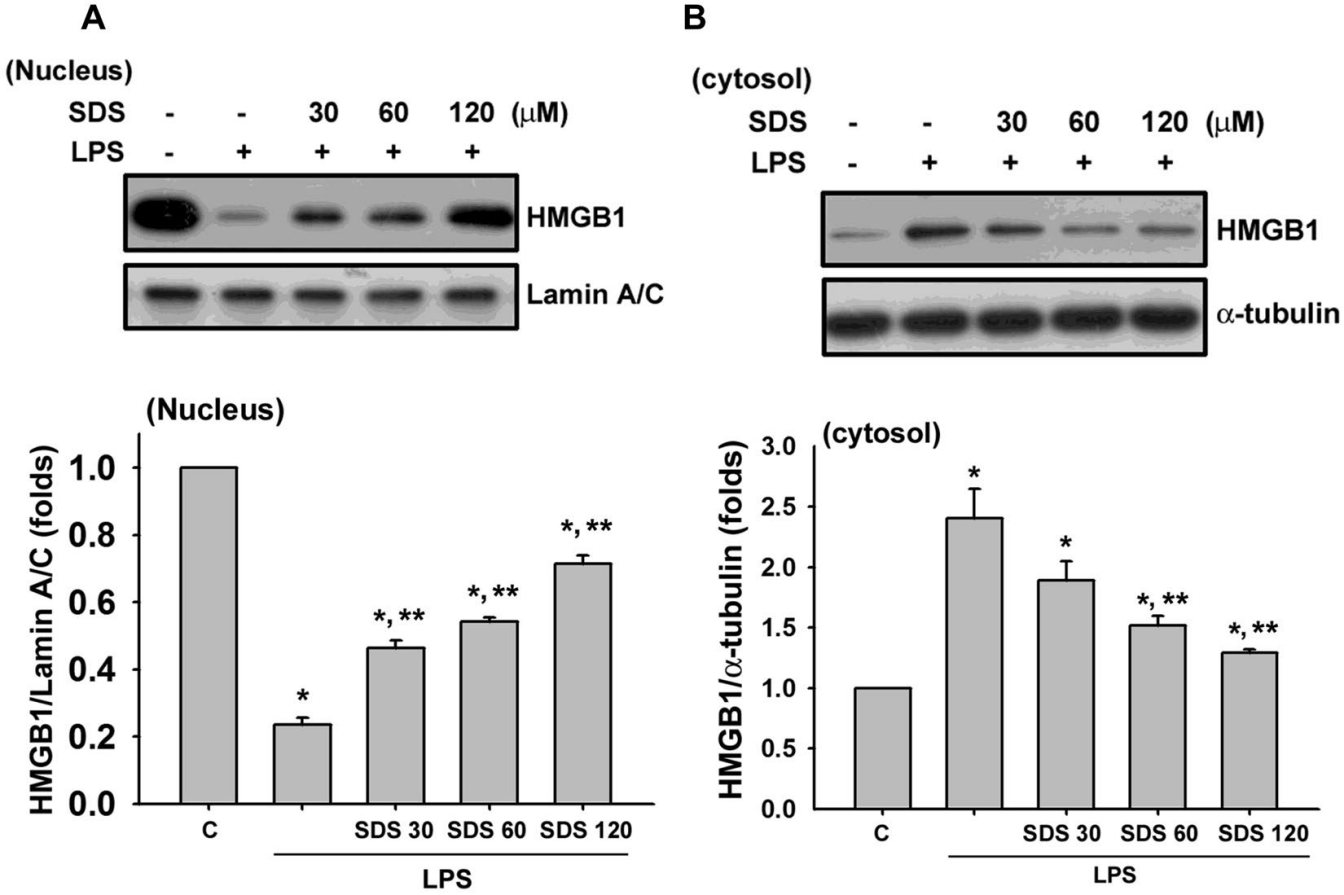
Effect of salidroside on the LPS-induced HMGB1 nucleocytoplasmic translocation in macrophages. RAW 264.7 macrophages were incubated with salidroside (30, 60, and 120 μM) for 1 h, and then treated with LPS (10 μg/ mL) for 24 h. The protein expressions of HMGB1 in both nucleus (**A**) and cytosol (**B**) were determined by Western blot. The quantification of protein expression was determined by densitometric analysis. Data are presented as means ± SEM for three independent experiments. *P < 0.05 as compared with control. **P < 0.05 as compared with LPS alone.

As shown in Fig. 1, salidroside at the concentrations of 30–120 μM did not affect the cell viability in RAW264.7 macrophages (Fig. 1A), but effectively and dose-dependently inhibited the LPS-induced increase of HMGB1 levels in cell medium (Fig. 1B). LPS treatment also markedly induced the iNOS protein expression (Fig. 1C) and NF-κB p65 phosphorylation (Fig. 1D), but decreased the SIRT1 protein expression (Fig. 1E) in macrophages, which could be significantly reversed by salidroside in a dose-dependent manner. Moreover, LPS significantly decreased the nuclear HMGB1 protein expression, but significantly increased the cytosolic HMGB1 protein expression in macrophages (Fig. 2). Salidroside significantly inhibited this LPS-induced HMGB1 nucleocytoplasmic translocation in macrophages in a dose-dependent manner (Fig. 2).

To clarify the relationship between SIRT1 and NF-κB signals, the siRNA-SIRT1 transfection and a selective NF-κB inhibitor pyrrolidine dithiocarbamate (PDTC) were used. As shown in Fig. 1F, the inhibitory effect of salidroside on LPS-induced NF-κB p65 phosphorylation could be effectively reversed by siRNA-SIRT1 transfection; however, PDTC could not affect the inhibitory effect of salidroside on LPS-decreased SIRT1 protein expression. These results indicate that SIRT1 is as an upstream signaling molecule of NF-κB in the present experimental situation.

**Figure 3:**
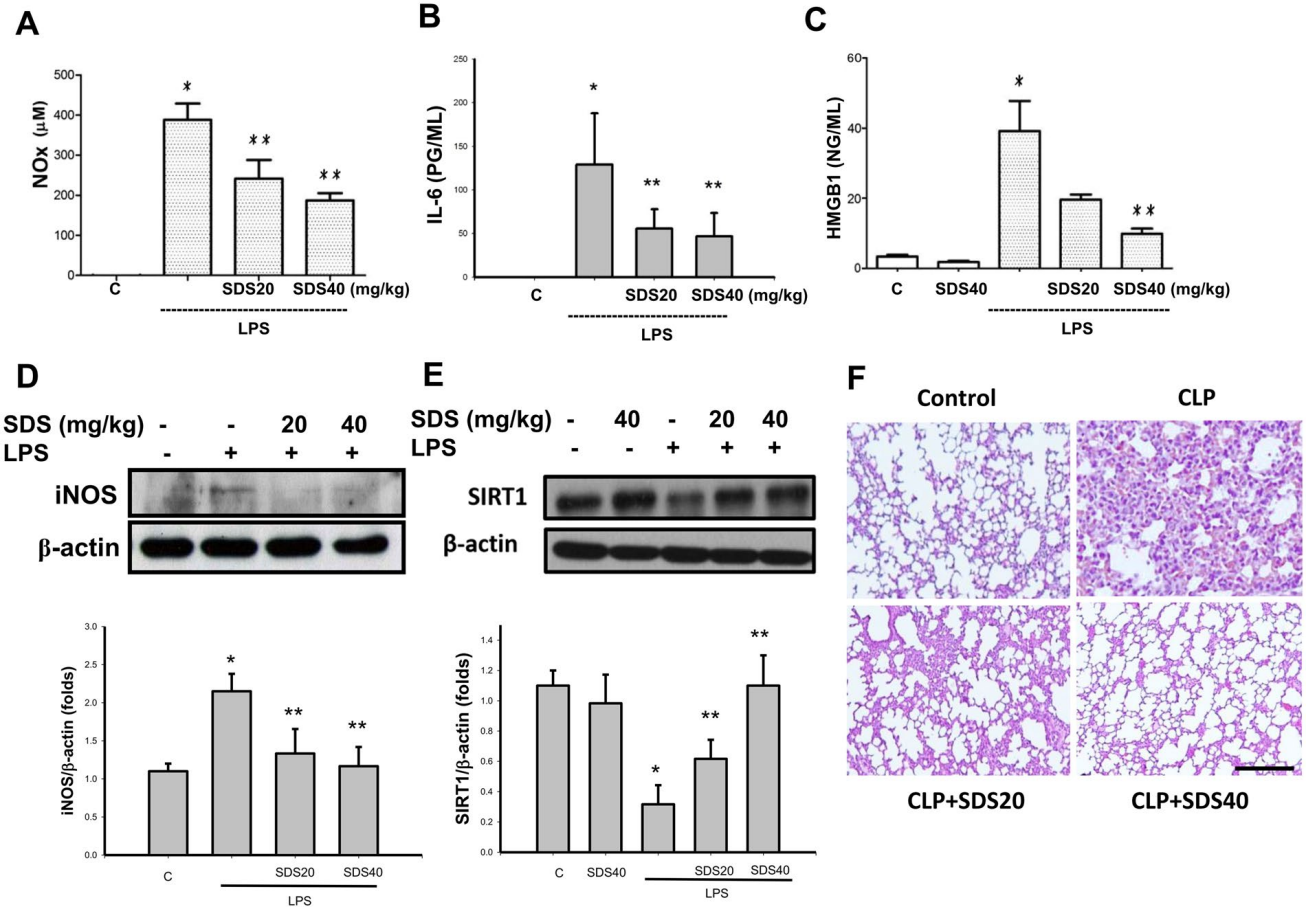
Salidroside inhibited inflammatory responses in a LPS-induced systemic inflammation mouse model. Mice were intraperitoneally injected with LPS (10 mg/kg) for 6 h (**A**), NOx production; (**D**) iNOS expression) or 24 h (**B**), IL-6 production; (**C**) HMGB1 productions; (**E**) SIRT1 expression; (**F**) lung histological change) in the presence or absence of salidroside (SDS; 20 and 40 mg/kg). The levels of serum IL-6 and HGB1 were determined by ELISA kits. The levels of serum NOx (nitrite/nitrate) were determined by nitrite/nitrate colorimetric assay kit. The protein expressions were determined by Western blot. Lung specimens stained with hematoxylin and eosin. Scale bar = 100 μm. Data are presented as means ± SEM (n = 6 mice per group). *P < 0.05 as compared with control (vehicle). **P < 0.05 as compared with LPS alone.

We next investigated the effects of salidroside on the inflammatory responses in LPS-induced systemic inflammation mouse model. Salidroside (20 and 40 mg/kg) treatment effectively decreased the serum NO (NOx, nitrite/nitrate; Fig. 3A), IL-6 (Fig. 3B), and HMGB1 (Fig. 3C) levels in mice subjected to LPS stimulation.Salidroside treatment could also inhibit the iNOS protein expression (Fig. 3D) and increase the SIRT1 protein expression (Fig. 3E) in the lungs of mice subjected to LPS. Moreover, we examined the lung specimens stained with haematoxylin and eosin for histological evaluation. The lung tissues from control mice showed minimal inflammation. There were the moderate pulmonary haemorrhage, interstitial edema, necrosis, congestion, and inflammatory cell infiltration were observed in the lungs of LPS-induced systemic inflammation mouse model, which could be reversed by salidroside treatment (Fig. 3F).

### Salidroside ameliorated sepsis-induced acute lung injury and lung lipid peroxydation and improves survival rate

**Figure 4:**
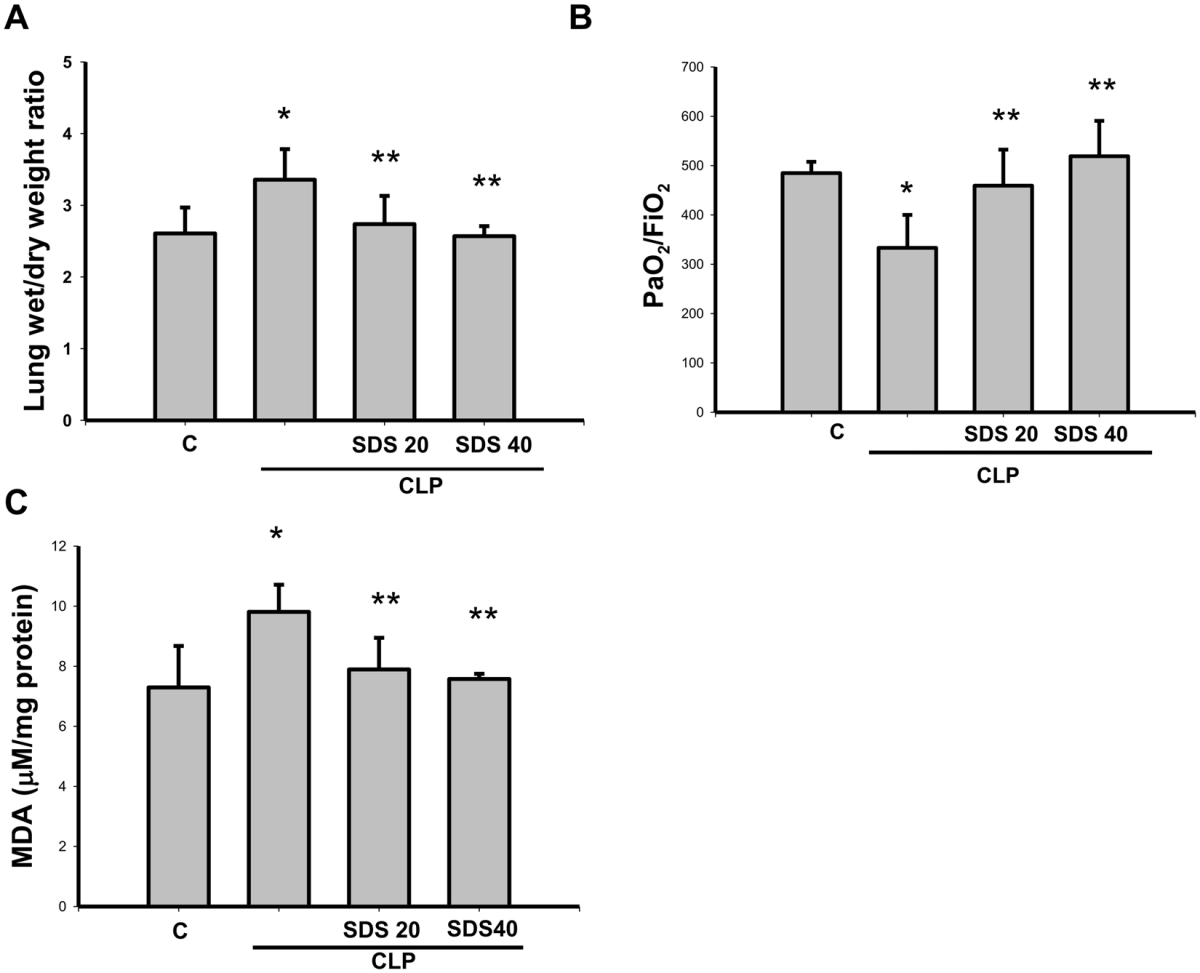
Salidroside attenuates sepsis-induced lung edema and lipid peroxidation and improves PaO_2_/FiO_2_ ratio. Sepsis was induced through cecal ligation and puncture (CLP). (**A**) Lung edema was measured as the wet/dry weight ratio in mice subjected to CLP for 24 h. Salidroside (SDS 20 or 40 mg/kg) was administered to mice 30 min after CLP. (**B**) The PaO_2_/FiO_2_ ratio was detected after CLP in mice with and without salidroside treatment. (**C**) Malondialdehyde (MDA, lipid peroxidation marker) levels were detected in the lungs of mice subjected to CLP for 24 h. Salidroside (SDS, 20 or 40 mg/kg) was given 30 min after CLP. All data are presented as means ± SEM (n = 6 mice per group). *P < 0.05 as compared with control (vehicle). **P < 0.05 as compared with CLP alone.

We next investigated the effects of salidroside on the inflammatory responses in another septic mouse model by CLP. CLP-induced lung edema was detected by an increase in the wet weight to dry weight ratio 24 h after the procedure (Fig. 4A). The PaO_2_/FiO_2_ ratio (an oxygenation index) also markedly decreased in mice subjected to CLP for 24 h (Fig. 4B). Salidroside (20 and 40 mg/kg) administered 30 min after CLP effectively reversed CLP-induced lung edema and improved the PaO_2_/FiO_2_ ratio in mice subjected to CLP (Fig. 4A and B). In addition, the lipid peroxidation in the lungs was evaluated in septic mice. MDA is a lipid peroxidation marker for assessing lipid peroxidation resulting from increased oxidative stress. As shown in Fig. 4C, the pulmonary MDA levels were markedly increased in CLP-induced septic mice, which were significantly reversed by salidroside treatment.

**Figure 5:**
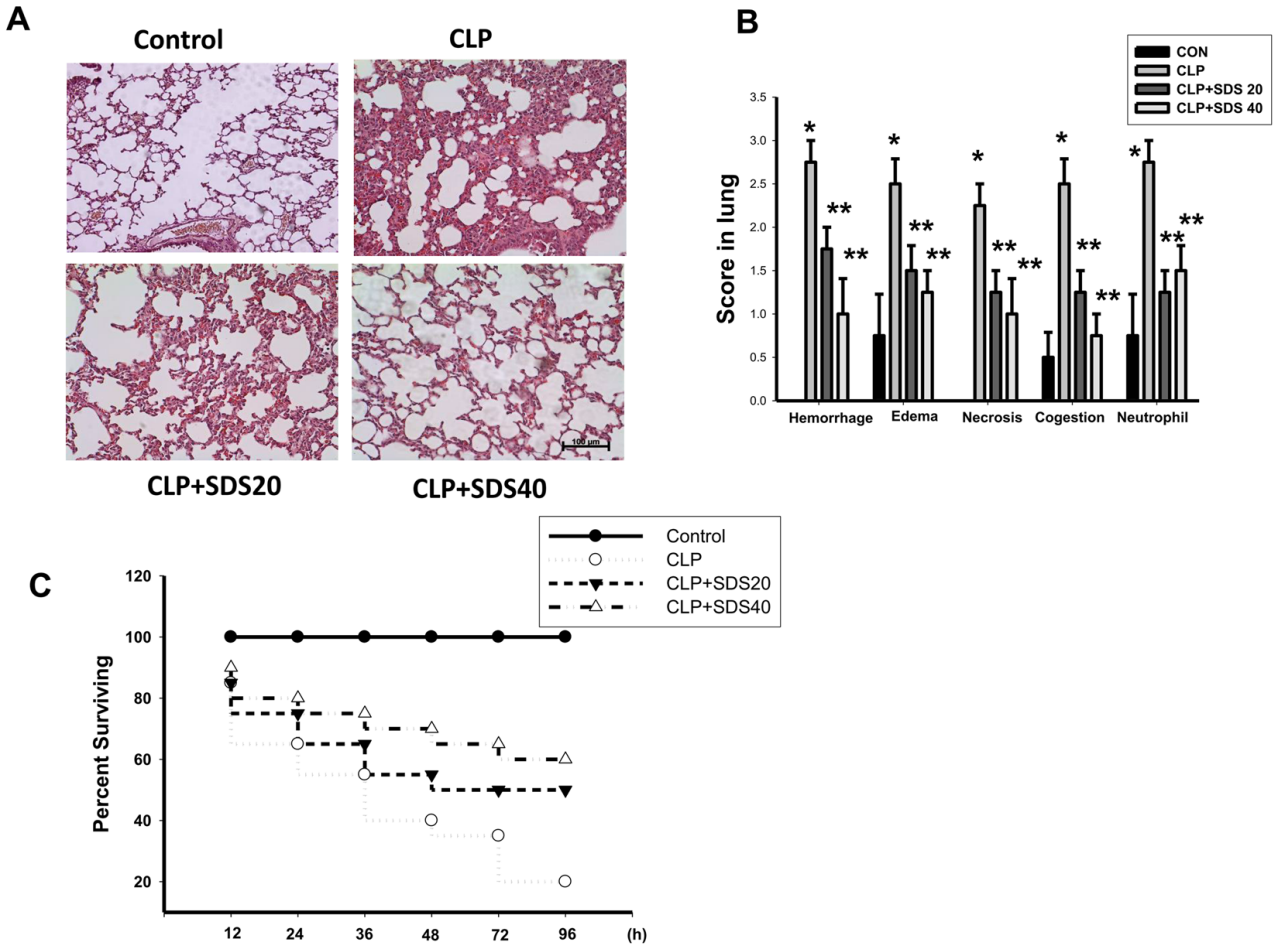
Salidroside attenuates sepsis-induced histological changes and ameliorates survival rate in mice. (**A**) Representative histological sections of lungs harvested 24 h after CLP application in mice with and without salidroside treatment. a. sham control mice; b. CLP-treated mice: the photograph shows marked inflammatory cell infiltration, interstitial edema, and vascular congestion; c. CLP-treated mice coupled with salidroside (20 mg/kg) treatment; d. CLP-treated mice coupled with salidroside (40 mg/kg) treatment. Lung specimens stained with hematoxylin and eosin. Scale bar = 100 μm. (**B**) The pathological score was shown. Data are presented as means ± SEM (n = 6 mice per group). *P < 0.05 as compared with control (vehicle). **P < 0.05 as compared with CLP alone. (**C**) Salidroside (SDS, 20 and 40 mg/kg) was administered to mice 30 min after CLP. Mortality was monitored twice daily for 96 h. Data are presented as the survival percentage of animals (n = 10 mice per group).

To determine the effects of salidroside on the CLP-related lung injury, we examined the lung specimens stained with haematoxylin and eosin for histological evaluation. The lung tissues from control mice showed minimal inflammation. The lung tissues from CLP-treated mice showed severe pulmonary hemorrhage, necrosis, congestion, inflammatory cell infiltrate, and diffuse alveolar septal thickening. CLP stimulation-induced histological changes in the lungs were effectively reversed by salidroside (20 and 40 mg/kg) treatment (Fig. 5A and B). The lung tissues from mice treated with CLP + salidroside 20 mg/kg or CLP + SDS 40 mg/kg showed less inflammatory cell infiltrate, congestion, and diffuse edema than CLP alone-treated mice. Moreover, salidroside treatment conferred protection against CLP-induced lethality, increasing the animal survival rate from 20 to 60% (Fig. 5C).

### Salidroside attenuated CLP-increased serum TNF-α, NO, and IL-6 levels and lung iNOS expression and NF-κB activation

**Figure 6:**
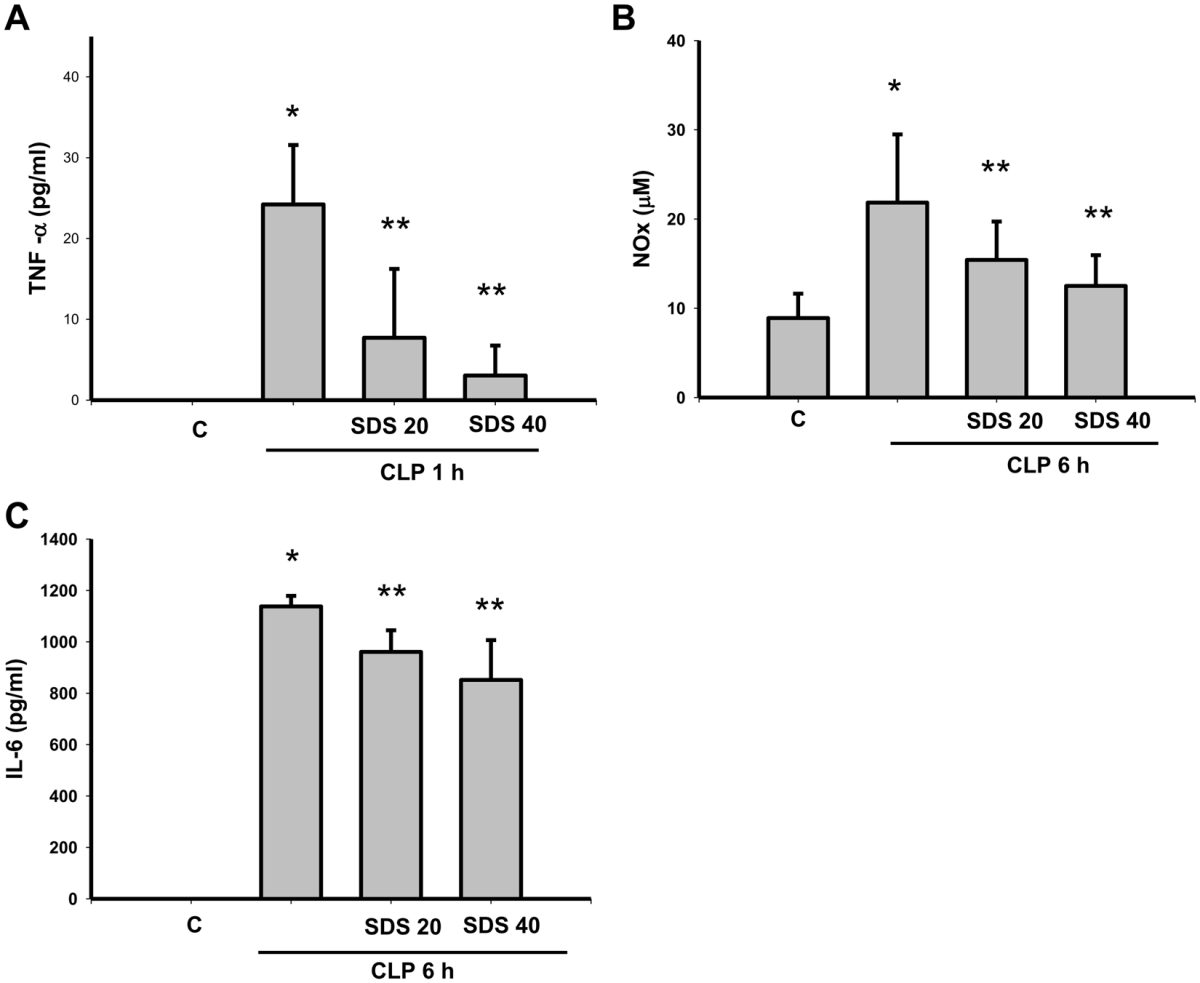
Salidroside decreases the serum levels of TNF-α, NO, and IL-6 in mice subjected to CLP. The serum levels of TNF-α, NO (NOx, nitrite/nitrate), and IL-6 were detected in mice subjected to CLP for 1 h, 6 h, and 24 h, respectively. Salidroside (SDS, 20 and 40 mg/kg) was injected 30 min after CLP. Data are presented as means ± SEM (n = 6 mice per group). *P < 0.05 as compared with control (vehicle). **P < 0.05 as compared with CLP alone.

Serum TNF-α (Fig. 6A), NO (nitrite/nitrate, NOx) (Fig. 6B), and IL-6 (Fig. 6C), levels were increased in mice subjected to CLP procedure. CLP-increased TNF-α, NOx, and IL-6 levels were significantly inhibited by salidroside (20 or 40 mg/kg) treatment administered 30 min after the CLP procedure (Fig. 6A–C).

**Figure 7:**
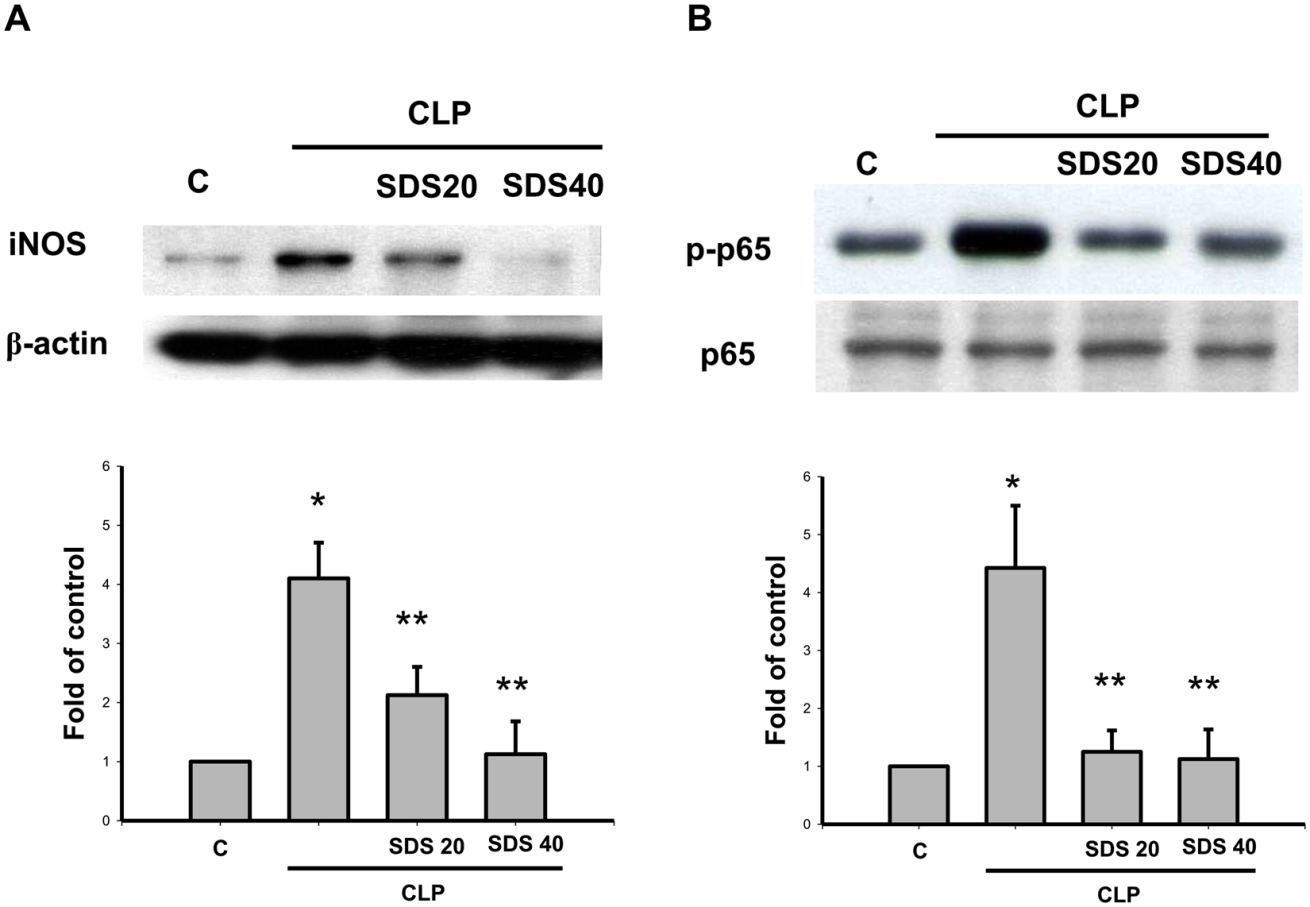
Salidroside attenuates iNOS protein expression and NFκB-p65 phosphorylation in the lungs of mice subjected to CLP. The iNOS (**A**) and phospho-NFκB-p65 (**B**) protein expressions in the lungs were detected in mice subjected to CLP administration for 6 h. Salidroside (SDS, 20 and 40 mg/kg) was administered 30 min after CLP. Lung samples were prepared and subjected to Western blotting for protein expression and quantified by densitometry. Data are presented as mean ± SEM (n = 6). *P < 0.05 as compared with control (vehicle). **P < 0.05 as compared with CLP alone.

Increases of iNOS protein expression (Fig. 7A) and NF-κB p65 phosphorylation (Fig. 7B) were observed in the lungs of mice subjected to CLP or LPS for 6 h. Salidroside (20 or 40 mg/kg) administered 30 min after CLP effectively reversed CLP-induced increases in iNOS protein expression (Fig. 7A) and NF-κB p65 phosphorylation (Fig. 7B).

### Salidroside inhibited HMGB1 nucleocytoplasmic translocation but upregulates SIRT 1 expression in the lungs of septic mice

**Figure 8:**
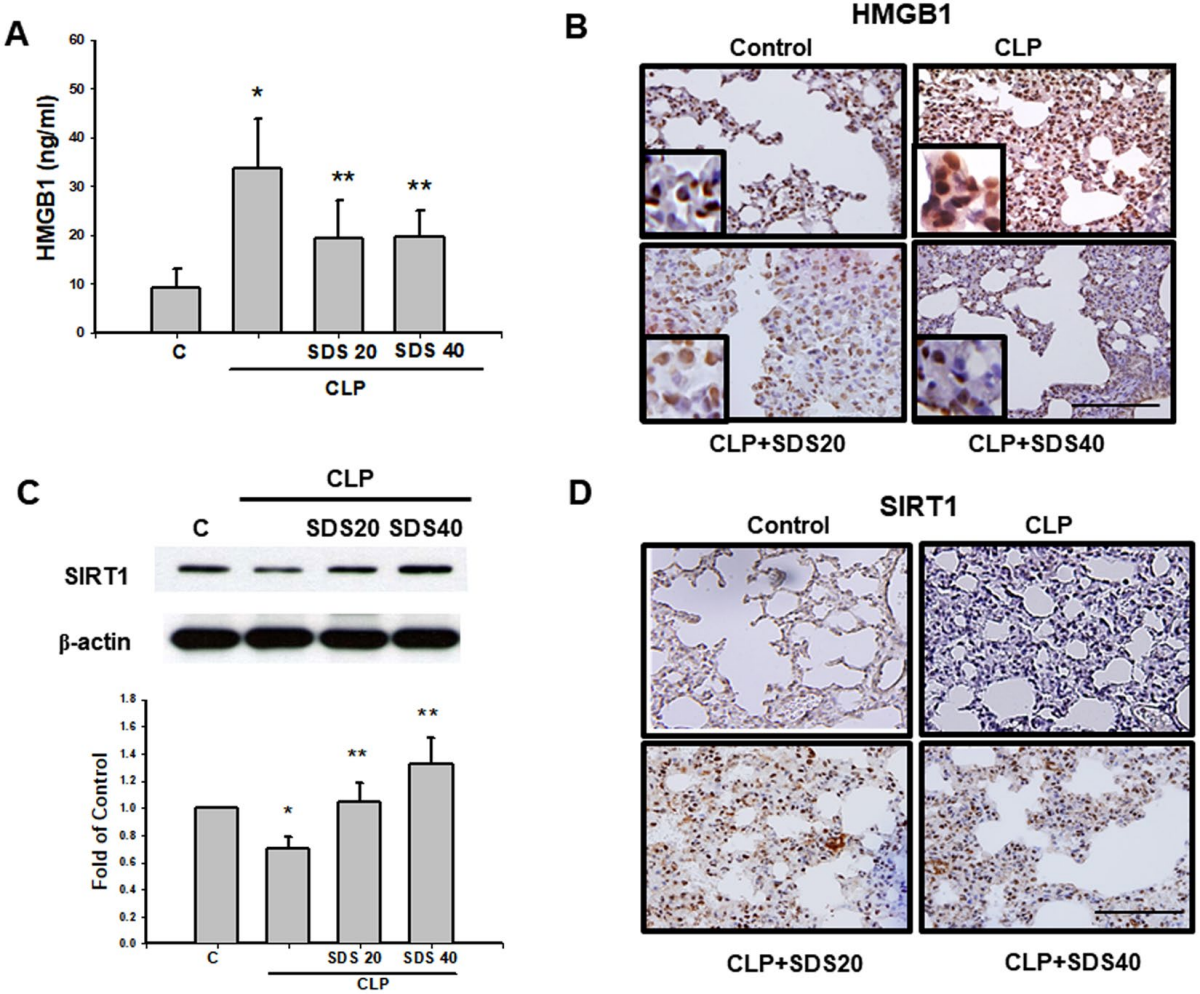
Salidroside attenuates HMGB1 levels in the sera and lungs and upregulates SIRT1 protein expression in the lungs of mice subjected to CLP. Sepsis was induced by CLP in the presence or absence of salidroside (SDS, 20 and 40 mg/kg), which was administered 30 min after CLP. (**A**) The serum levels of HMGB1 were detected in mice subjected to CLP for 24 h. Data are presented as mean ± SEM (n = 6). *P < 0.05 as compared with control (vehicle). **P < 0.05 as compared with CLP alone. (**B**) HMGB1 protein expression in the lung was determined by immunohistochemistry in mice 24 h after CLP. Salidroside (SDS, 20 and 40 mg/kg) was administered 30 min after CLP. The results shown are representative of four independent experiments. Scale bar = 100 µm. An insert of larger scale image for HMGB1 nucleocytoplasmic translocation was shown in each indicated figures (blue stain for nucleus; dark brown stain for HMGB1). The protein expression of SIRT 1 in the lungs was determined by Western blotting (**C**) and immunohistochemistry (**D**) 24 h after CLP. Data are presented as means ± SEM (n = 6). *P < 0.05 as compared with control (vehicle). **P < 0.05 as compared with CLP alone.

CLP-induced increased serum HMGB1 levels were significantly inhibited by salidroside (20 or 40 mg/kg) treatment administered 30 min after the CLP procedure (Fig. 8A). HMGB1 stores in the nucleus and translocates to the cytosol activated by bacterial LPS (Bonaldi *et al*. 2003). HMGB1 nucleocytoplasmic translocation was observed in the lungs of mice 24 h after CLP (Fig. 8B). Salidroside (20 and 40 mg/kg) treatment in mice 30 min after CLP effectively reversed the HMGB1 nucleocytoplasmic translocation in the lungs (Fig. 8B). Moreover, SIRT1 protein expressions determined by immunoblotting and immunohistochemistry in the lungs were markedly reduced in mice subjected to CLP (Fig. 8C). Salidroside (20 or 40 mg/kg) effectively upregulated the SIRT1 protein expressions in the lungs of septic mice (Fig. 8D).

## Discussion

In the present study, we used the LPS-induced systemic inflammation mouse model or CLP-induced murine sepsis model to investigate the therapeutic role of salidroside in sepsis-induced acute lung injury. Salidroside attenuated the lipid peroxidation in lung tissues and the secretions of inflammatory mediators (TNF-α, NO, and HMGB1) under LPS or CLP- induced systemic inflammation or septic shock. Sepsis increased NF-κB activation and HMGB1 nucleocytoplasmic translocation and iNOS protein expression, but decreased SIRT1 protein expression in lung tissues. Salidroside significantly and effectively upregulated SIRT1 expression, inhibited the inflammatory cascade reaction, decreased the NF-κB activation and HMGB1 nucleocytoplasmic translocation, reduced the severity of acute lung injury, and improved the survival rate during sepsis. These findings suggest that salidroside exerts the therapeutic effect on acute lung injury in a septic shock mouse model.

NF-κB is pivotal in inflammatory responses because it induces the secretion of proinflammatory cytokines (eg. IL-6, IL-1β, and TNF-α) and iNOS (Hayden and Ghosh 2008). In response to various stimuli, such as cytokines, DNA-damaging agents, and bacterial wall or viral proteins, the IκB is dissociated, and the activated transcription factor translocates to the nucleus, thus inducing a large number of target genes involved in cell growth, apoptosis, cell adhesion, and inflammation^20^. The NF-κB system is critical in regulating the innate immunity responses in host tissues^21^. In addition, HMGB1, a late mediator of inflammation during sepsis, is essential in the responses of sepsis-induced injury. HMGB1 can be extensively modified (eg. hyperacetylated, in LPS-activated monocytes) and released, contributing to inflammatory responses^22,23^. Nucleocytoplasmic HMGB1 translocation increases in acute lung injury, and the inhibition of HMGB1 secretion attenuates systemic inflammatory response syndrome and sepsis-induced organ injury^19,23^. Therefore, inhibiting HMGB1 translocation and release might protect against sepsis-induced acute lung injury and may offer a wider therapeutic window for sepsis. In the present study, the activation of NF-κB coupled with proinflammatory cytokines secretion and the increase of HMGB1 protein expression/nucleocytoplasmic translocation were observed in the lungs during sepsis-induced acute lung injury, which could be effectively reduced by salidroside treatment. These results suggest that salidroside is capable of ameliorating the early phase and late phase of sepsis-related inflammatory responses mediated by NF-κB-regulated proinflammatory cytokines and HMGB1, respectively.

SIRT1 is known to specifically remove the acetyl groups from lysine residues and causes histone and non-histone protein deacetylation^10^. Previous studies have demonstrated that SIRT 1 plays a principal role in modulating the development and progression of inflammation and exerts anti-inflammatory effects by regulating the production of proinflammatory cytokines^24–26^. SIRT1 is critical in silencing the NF-κB-driven transactivation of TNF-α and IL-1β genes and thus is essential in the termination of the inflammatory response^26,27^. Ablation of SIRT1 in macrophages has been shown to render the NF-κB hyperacetylation and then enhance the activation of proinflammatory genes^28^. SIRT1 activators have been found to inhibit LPS-induced inflammatory responses and cytokines secretion in macrophages in a SIRT1-dependent manner^29^. These findings provide a rationale for the use of SIRT1 activators in therapy against inflammatory diseases. Moreover, increase in lung ICAM-1 and HMGB1 expression in SIRT1-deficient mice aggravated lung injury after sepsis^11^. SIRT1 siRNA transfection has been demonstrated to increase the cytoplasm HMGB1 levels and decrease the nucleus HMGB1 levels in the human liver cells, indicating a reduction in the SIRT1-mediated suppression of HMGB1 translocation^21^. Posttranslational modification of HMGB1 is a critical step in regulating the release of this protein during inflammatory responses^30^. Upregulation of SIRT1 modulates the status of HMGB1 acetylation, which in turn is critical in the cellular response to inflammation through deacetylation-mediated regulation of HMGB1 release^22^. Attempts to correct HMGB1 intracellular distribution through SIRT1 activation and improve nuclear retention during stress appear to be rational. In the present study, salidroside treatment reversed both sepsis-induced downregulation of SIRT1 expression and increased proinflammatory cytokines (TNF-α and IL-6) secretion. We also examined the effect of salidroside on the LPS-induced NF-κB and HMGB1 production in macrophages and found that salidroside was capable of inhibiting the NF-κB and HMGB1 production in response to LPS, and it may be related to the upregulation of SIRT1. Salidroside enhanced SIRT1 expression and consequently inhibited HMGB1 acetylation and nucleocytoplasmic translocation.

HMGB1 nucleocytoplasmic translocation is extensively associated with HMGB1 acetylation in activated macrophages by the stimulation of LPS or TNF-α^7^. This acetylation-associated translocation is mediated by chromosome region maintenance 1 (CRM1), a nuclear exportin^31^. The inhibition for HMGB1 acetylation or CRM1 has been explored as a potential suppression of HMGB1 nucleocytoplasmic translocation^7,32^. A previous study has also reported that the acetylation on HMGB1 localization correlated with the deacetylase activity of SIRT1, indicating that SIRT1 interacts with HMGB1 and deacetylates it and thereby prevents its release^22^. In the present study, we found that salidroside could activate SIRT1 protein and the inhibition of HMGB1 nucleocytoplasmic translocation. We, therefore, infer that salidroside suppresses HMGB1 nucleocytoplasmic translocation by the activation of SIRT1 deacetylase activity. Moreover, AMP-activated protein kinase (AMPK)-dependent mechanism has been shown to be involved in protecting against LPS-induced organ injury^32^. It has been found that the activation of AMPK increases the SIRT1 expression in macrophages^33^. A previous study has also indicated that the protective effect of salidroside on neuron may be mediated by AMPK/SIRT1 pathway^34^. On the other hand, salidroside has been found to be a novel pharmacological inhibitor of estrogen receptor-α (ERα)/prolyl-hydroxylase domain 3 (PHD3) axis for therapeutic angiogenesis in hind-limb ischemia diseases^35^. The ERα might be the potential putative target of salidroside for neovascularization. However, the real cellular target of salidroside for SIRT1-regulated NF-κB activation inhibition and HMGB1 nucleocytoplasmic translocation inhibition during sepsis still needs to be clarified in the future.

In this study, we found that single dose salidroside treatment attenuated CLP or LPS-induced severe systemic inflammation in mice administered intraperitoneally with a single dose 30 min after CLP or LPS stimulation. According to the previous studies, the mean elimination half-life (t1/2) of salidroside in rats following intravenous or oral administration was around 0.5 h or 1.1 h, respectively^36,37^. However, the pharmacokinetics after intraperitoneal administration in mice has not yet been reported. In addition, other studies also showed that salidroside given by one dose of intraperitoneal injection was effective in the CLP model or traumatic head injury model^18,38^.

The present study showed that salidroside treatment significantly improved the survival of sepsis and suppressed proinflammatory cytokines challenged by LPS or CLP stimulation, which is exactly consistent with the previous findings^17,18,39–41^. Nevertheless, there are some unique findings in our study. Systematic inflammation response is considered a hallmark feature of sepsis. In the present work, the early phase cytokines including TNF-α, IL-6, and NOx and a late lethal mediator HMGB1 were effectively inhibited after salidroside administration. HMGB1 released from activated macrophages is an endogenous danger signal to augment inflammatory responses in sepsis. This may contribute to the lasting and severe inflammatory reactions and miserable results. According to our review of relevant literatures, the effects of salidroside on late phase mediator of HMGB1 in severe sepsis and sepsis-induced acute lung injury models were seldom reported in these previous studies. Furthermore, we elucidated that salidroside prevented the HMGB1 nucleocytoplasmic translocation and HMGB1 release during sepsis via a SIRT1-mediated signaling pathway.

In conclusion, the increase in the production of early and late proinflammatory mediators in sepsis likely leads to mortality and acute lung injury. Salidroside is one of the major phenolic glycosides in *Rhodiola* ^13^. The present study showed that salidroside was promising as a therapeutic agent of septic mice. Salidroside reduced the production of pro-inflammatory cytokines (TNF-α and IL-6) through a SIRT1-mediated inhibition of NF-κB activation pathway in the early sepsis phase. In the late sepsis phase, salidroside protected against sepsis-induced acute lung injury through the SIRT1-mediated HMGB1 nucleocytoplasmic translocation pathway. Moreover, Salidroside may be a potential therapeutic agent for treating sepsis-induced acute lung injury and mortality. Further studies are needed to clarify the detailed molecular mechanisms of salidroside on sepsis therapy and explore whether it is a new strategy for clinical management of sepsis.

## Methods

### Cell Cultures

The mouse monocyte/macrophage cell line RAW264.7 (ATCC-TIB71) was used. Cells were cultured in DMEM medium (Gibco, Grand Island, NY. USA) supplemented with 2 mM glutamine, antibiotics (100 U/ml of penicillin A and 100 U/ml of streptomycin), and 5% heat-inactivated fetal bovine serum (Gibco) and maintained in a 37 °C humidified incubator containing 5% CO_2_. In some experiments, the siRNAs against SIRT1 and control siRNA were commercially obtained from Invitrogen. RAW264.7 cells were transfected with siRNAs (60 nM) using RNAimax (Invitrogen) as described by the manufacturer’s instruction.

### Animal model of sepsis

ICR male mice (20–25 g), provided by the Laboratory Animal Centre of the College of Medicine, National Taiwan University (Taipei, Taiwan), were used in all experiments. All animal studies were approved by the ethical review committee of College of Medicine, National Taiwan University, and were carried out in accordance with regulations of Taiwan and NIH guidelines on the care and welfare of laboratory animals. All animals were treated humanely and with regard for alleviation of suffering. Mice were maintained under pathogen-free conditions with 12:12 h light–dark cycle. Endotoxemia was induced in mice by intraperitoneal (i.p.) injection of bacterial endotoxin (LPS, *E. coli* 055:B5-, Sigma), 10 mg/kg. Moreover, sepsis was also induced through cecal ligation and puncture (CLP; Weng *et al*. 2011). Mice were fasted overnight before the surgical procedure. Mice were anaesthetised using an intraperitoneal pentobarbitol (30 mg/kg) injection. Subsequently, laparotomy was performed, and the cecum was exposed. The cecum was ligated below the ileocecal valve and punctured twice using an 18-gauge needle, and the bowel contents were extruded. The cecum was returned and the abdominal cavity was closed. The procedure, except CLP, was repeated for the sham mice. Salidroside (KinderChem, Hangzhou, China) was dissolved in 0.9% saline (10 mg salidroside in 1 ml saline; 10 μg/μl). Salidroside (20 and 40 mg/kg, about 60–120 μl per mouse) was intraperitoneally administered 30 min after the surgical procedure. The control mice were administrated with an equal volume of vehicle.

### Measurement of PaO_2_/FiO_2_ ratio

Mice were intraperitoneally anesthetized with pentobarbital injections 24 h after CLP in the presence or absence of salidroside. The carotid arteries were cannulated, and the arterial blood samples were collected for PaO_2_ analysis. The oxygenation index was expressed as PaO_2_/FiO_2_.

### Lung edema measurement and histological evaluation

Mice were sacrificed under pentobarbitol anaesthesia and the lungs were excised. All extrapulmonary tissues were cleared, weighed (wet weight), dried for 48 h at 60 °C, and weighed again (dry weight). Lung edema was expressed as the ratio of the wet weight to the dry weight. For histological evaluation, the lungs were fixed using an intratracheal instillation combined with 10% neutral phosphate-buffered formalin at a pressure of 20 cm H_2_O for at least 72 h. Pulmonary lobe slices (thickness, 2–3 mm) were embedded in paraffin. The paraffin-embedded sections (3 μm) were stained using haematoxylin and eosin. Pulmonary alterations were scored by an experienced pathologist in blinded fashion, using the grading system involved measurements of grading system involved measurements of haemorrhage, interstitial edema, necrosis, vascular congestion, and inflammatory cells infiltration, each on a scale of 0–3.

### Immunohistochemical analysis

Lung tissues were immediately fixed in 4% paraformaldehyde and embedded in paraffin, and 3-µm-thick sections were prepared. The sections were deparaffinized, and the endogenous peroxidase activity was blocked by incubation with 3% H_2_O_2_ (10 min). The blocked sections were incubated with anti-HMGB1 (dilution, 1:1000, Abcam, Cambridge, MA, USA) and anti-SIRT1 antibodies (dilution, 1:1000, Proteintech, Chicago, IL, USA). Primary antibody binding was visualised using 3,3′-diaminobenzidine (10 min). After development for antibody labelling, the sections were counterstained using Mayer haematoxylin and mounted.

### TNF-α, IL-6, HMGB1, and Nitrite/Nitrate assays

ELISA kits were used to assay the levels of TNF-α (R&D Systems, Minneapolis, MN, USA), IL-6 (eBioscience, San Diego, CA, USA), and HMGB1 (Shino-Test, Tokyo, Japan) in the mouse serum or cell media after CLP or LPS stimulation. Serum nitrite levels were determined using the nitrite/nitrate colorimetric assay kit (R&D Systems).

### Lipid peroxidation assay

Blood samples were collected from the peripheral vessels of anesthetized mice. Whole blood was centrifuged at 3000 rpm for 10 min, and the plasma was obtained and assayed immediately using the lipid peroxidation [malondialdehyde (MDA)] assay kit (Calbiochem, Merck KGaA, Darmstadt, Germany). Absorbance at 586 nm was measured using an ELISA microplate reader.

### Western blot analysis

Total proteins containing 30–80 μg were separated on 8% SDS‐polyacrylamide mini gels and transferred to nitrocellulose membranes. After blocking, the blots were incubated with antibodies for NF-κB p65, iNOS, lamin A/C, α-tubulin (Santa Cruz Biotechnology, Dallas, TX, USA), and SIRT1 (Abcam) in PBS or Tween 20 for 1 h, followed by two washes in PBS or Tween 20, and subsequently incubated with horseradish peroxidase-conjugated goat anti-mouse IgG for 30 min. β-Actin was the control for sample loading and integrity. The antibody-reactive bands were revealed using an enhanced chemiluminescence kit (Amersham, Pittsburgh, PA, USA), and the bands were exposed to a Kodak radiographic film. The amount of polypeptide was quantitated by integrated densitometric analysis of the film (Kodak Gel Logic‐100 Imaging System).

### Statistics

Data are expressed as means ± SEM. Statistical analysis was performed using one-way analysis of variance followed by the Dunnett test for each paired experiment. For mortality test, the Kaplan–Meier plots were used and a statistical assessment by log-rank test was performed. *P* < 0.05 was considered statistically significant.
